# Supplementary material for: Effectiveness evaluation of connected and automated vehicles’ driving loop: Node weights and driving reliability
Source: PLoS One. 2025 Jun 23;20(6):e0325837. doi: 10.1371/journal.pone.0325837 (PMC12184932; doi:10.1371/journal.pone.0325837)
Supplement: Attachment — Submitted filename: Response to Reviewers.docx [file pone.0325837.s006.docx]

Dear editors and reviewers，

We are very grateful for your constructive comments and suggestions for our manuscript entitled "***Effectiveness Evaluation of Connected and Automated Vehicles' Driving Loop: Node Weights and Driving Reliability***" (Manuscript Number: **PONE-D-25-02537**). Your comments are very valuable and helpful for improving our manuscript. We have carefully revised the manuscript and provided point-by-point response below.

Reviewer 1

Comments 1: The research lacks focus, and the paper's organization is described as messy. As a result, the contributions are not clear.

Response 1: Thank you very much for your valuable suggestions. Regarding the issues you mentioned, we have redrafted the title and modified it to "***Effectiveness Evaluation of Connected and Automated Vehicles' Driving Loop: Node Weights and Driving Reliability***". At the same time, we have also reorganized the introduction part, elaborated in detail on the research progress of vehicle network modeling, vehicle efficiency evaluation, and node importance, and deeply explored the deficiencies of existing methods. In the "Contributions of This Paper" section, we have rewritten the content and clearly expounded the research objectives and innovative points. In the methods section, we have modified the titles of some chapters, taking the paper title "***Effectiveness Evaluation of Connected and Automated Vehicles' Driving Loop: Node Weights and Driving Reliability***" as the main line. Meanwhile, we have added a flowchart for calculating the node weights and a flowchart of the entire research process. The former presents in detail the specific steps for calculating the node weights by using the Interpretive Structural Modeling (ISM), complex network theory, and taking driving reliability into account. The latter clearly demonstrates the whole process from the construction of the network model, to the analysis of node importance using various methods, the evaluation of driving reliability, and finally to the completion of the efficiency evaluation of the network model through the improved information entropy method.

We sincerely hope that these revisions can make the structure of the paper clearer and the logic more rigorous, thus facilitating readers' understanding. The following are the revised paragraphs and the added visual graphics:

**1 Introduction**

With the continuous advancement of technology, including the rapid development of sensor technology, communication technology, and artificial intelligence, the Connected and Automated Vehicles (CAVs) have emerged as a crucial direction for the transformation and upgrading of the automotive industry. Additionally, the effectiveness evaluation of CAVs during the driving process has emerged as one of the prominent topics in automotive research. Vehicle effectiveness evaluation is a process that comprehensively and systematically analyzes and assesses a vehicle's capability, efficiency in accomplishing various tasks, and the extent to which goals are achieved in specific usage scenarios. With the continuous evolution of systems science and complex network theory, their applications in effectiveness evaluation are becoming more and more extensive.

In previous research on vehicle network modeling, Yang L et al. [1] proposed a vehicle network model based on the peer-to-peer (P2P) network, aiming to enhance the network's fault tolerance and ensure the stability of the network system. Ma J et al. [2] developed a stochastic electric vehicle network model that incorporated environmental costs and proposed an algorithm for solving this network model. Tu Q et al. [3] proposed a reliability-based equilibrium model for electric vehicle networks, which was employed to predict the traffic flow patterns in the road networks of both electric vehicles and gasoline vehicles. Xu Q et al. [4] constructed and validated a hybrid traffic network model in which human-driven vehicles and CAVs coexisted.

Regarding the research on vehicle effectiveness, Zhao J et al. [5] established an overall subjective evaluation model associated with three objective indicators, leveraging the Probabilistic Neural Network (PNN) to assess the dynamic performance of vehicles. Researchers such as Wang B [6] endeavored to utilize the characteristic indicators of wheels to assess the stability performance of vehicles. Huang W L et al. [7] deliberated on the task-specific performance evaluation model of Unmanned Ground Vehicles (UGVs) applicable to the annual Intelligent Vehicle Future Challenge (IVFC) competition. Wang W et al. [8] proposed a novel quantitative comprehensive performance evaluation method for autonomous vehicles, which assesses autonomous vehicles quantitatively across four aspects: driving safety, riding comfort, intelligence, and efficiency. Park J Y et al. [9] devised a control algorithm for the torque vectoring system to enhance the handling performance of green vehicles and assessed the vehicle dynamics performance, thereby boosting controllability and stability.

Node weights can be used to measure the importance of nodes. The discourse on the significance of nodes in the network model can be primarily categorized into three types: First, the assessment of the significance of network nodes [10,11]; Second, the investigation taking into account the significance of nodes [12,13]; Third, the identification of crucial nodes in the network based on the significance of nodes [14,15]. When quantifying the significance of nodes in the network model, studies can be conducted from the viewpoints of the local network [16,17], the global network [18,19], and the integration of the two [20,21]. When exploring the significance of nodes by integrating the global and local aspects of the network, Yu Jintao et al. comprehensively take into account the outgoing and incoming intensities of nodes. They quantify the local significance of nodes via the information interaction intensity of a single node, while utilizing the node efficiency to quantify the global significance of nodes.

In conclusion, the majority of prior research endeavors concentrated on the network modeling of traditional vehicles or electric vehicles. Moreover, with respect to effectiveness evaluation, it was predominantly conducted from a microscopic viewpoint to evaluate specific vehicle performance aspects during driving, such as stability and dynamic performance. Few researchers have comprehensively considered the significance of nodes from both the global and local perspectives of the driving network for CAVs, nor have they accounted for the reliability of the driving system itself, leading to incomplete evaluation outcomes. The construction and optimization of network models are currently a research focal point, and they also represent a complex and challenging process [22,23]. The network of CAVs is a heterogeneous and open system, characterized by a complex network structure [24,25]. Moreover, numerous aspects of this system are uncertain.

The objective of this paper is to construct a network model of the driving loop for CAVs, optimize system performance, and enhance driving safety and reliability through scientific effectiveness assessment. Based on this, this paper innovatively proposes to construct a network model for CAVs grounded in the OODA loop. Simultaneously, the integrated model of the Interpretive Structural Model (ISM) and complex network theory is employed to measure the node network weights by incorporating both global and local perspectives. Additionally, driving reliability is considered through the fuzzy evaluation method, and the node weights that account for driving reliability are comprehensively determined. Finally, the effectiveness of the CAVs driving loop network model is evaluated by enhancing the information entropy model. This approach lays a solid foundation for in-depth research on the driving process of CAVs by constructing a driving loop network system, abstracting key module nodes including perception, information, decision, execution, and target, and clearly depicting the mutual influence relationships among nodes via directed edges. In the aspect of effectiveness evaluation, by comprehensively applying the analysis of node weights and the enhanced information entropy model, considering both the global and local characteristics of the network and driving reliability, this approach enables accurate assessment of the driving loop's effectiveness, thereby facilitating the establishment and continuous optimization of the network structure of the driving model for CAVs.

**The added flowchart:**

Fig. 5. Flowchart of Node Weights of the Driving Loop Network Model of CAVs.

Fig. 6. Flowchart of the Effectiveness Evaluation of the Driving Loop Network System of CAVs.

Comments 2: The abstract should be revised to better highlight the novelty of the work. Clearly articulating the innovative aspects of this study will help engage readers and set the stage for the detailed discussion that follows.

Response 2: Thank you very much for your valuable comments on the abstract of the paper. We fully agree with the view that the abstract should better highlight the innovation of the work. After carefully studying your feedback, we immediately revised the abstract. The following is the revised abstract.

**Abstract:** As an emerging development trend in the automotive industry, the construction of the network model and the effectiveness evaluation of the driving loop for Connected and Automated vehicles (CAVs) are of significant importance. The objective of this paper is to construct a network model of the driving loop for CAVs and evaluate the effectiveness of the model, thereby optimizing system performance and enhancing driving safety and reliability. In this study, by integrating the driving process of CAVs and introducing the concept of the Observation, Orientation, Decision, and Action (OODA) loop, a network model of the driving loop for CAVs was established, enabling effective modeling of the complex driving process. For effectiveness evaluation, a method is proposed. This method measures the importance of nodes using the Interpretive Structural Model (ISM) and complex network theory, considers driving reliability through the fuzzy evaluation method, and comprehensively determines the node weights of the network model. Subsequently, by utilizing the node weights to enhance the information entropy model, a scientific evaluation of the CAVs' driving loop effectiveness is achieved. Through comparisons and validations across several scenarios, it has been demonstrated that this method can be effectively applied to the planning, modeling, evaluation, and optimization of CAVs network models.

Comments 3: The quality of the figures is low and the images are unclear. Enhancing the clarity, resolution, and overall presentation of the figures will make them more effective in supporting and illustrating the manuscript's content.

Response 3: Thank you for your kind suggestion. In response, we have optimized the clarity, resolution and overall presentation of the charts.

Comments 4: It is suggested to use figures to visualize the steps involved in using the ISM method and the fuzzy evaluation method. Visual aids like these can greatly enhance understanding and accessibility of the methodologies employed.

Response 4: We are extremely grateful to you for pointing out the deficiencies in the visualization of the methods in the paper. Upon receiving your feedback, we promptly carried out optimization of this part, aiming to comprehensively enhance the clarity and intuitiveness of the method description.

We have drawn a detailed flowchart covering the entire research process, which is a schematic diagram of the overall process from the construction of the CAVs network model to the final efficiency evaluation. This diagram links together all the key stages from the construction of the network model of CAVs to the efficiency evaluation of the driving system. Each link is presented as a clear module in the diagram, and arrows are used to indicate the flow direction, supplemented by necessary textual annotations, making the research process clear at a glance.

In addition, for the key link of calculating the node weights, we have specially drawn a flowchart to meticulously display the implementation steps of methods such as Interpretive Structural Modeling (ISM), complex network theory, and driving reliability. The logical relationships between each step are clear and obvious, allowing readers to quickly grasp the principles and processes of calculating the node weights.

Through this series of flowcharts, not only can readers easily understand the research methods of the paper, but also the logic and professionalism of the paper have been further enhanced. Once again, thank you for your valuable suggestions, and we hope that the revised paper will meet with your approval.

Comments 5: The description of the data and settings for the case study is missing or unclear.

Response 5: Thank you for pointing out the problems in the description of data and settings in the case study of the paper. Here is a detailed response to your questions: The data in this paper does not come from external existing datasets, but is gradually generated during the research process of the paper.

At the beginning of the research, we deeply analyzed the driving process of CAVs, combined it with the cyclic process of the Observation, Orientation, Decision, and Action (OODA) loop, and constructed the driving loop network model of CAVs. At this stage, based on the actual perception, decision, execution and other links of CAVs, we defined perception-type, information-type, decision-making-type, execution-type and target-type nodes, and used directed edges to represent the mutual influence relationships between nodes. The data was generated during the process of determining the node types and the connection relationships of the edges.

Subsequently, in order to analyze the node weights of the driving loop network model of CAVs, we applied the combined model of Interpretive Structural Modeling (ISM) and complex networks. In this step, based on the adjacency matrix, we generated the reachability matrix, divided the network hierarchy, and then determined the hierarchical weights of each node to obtain the global weights of the nodes. At the same time, with the help of complex network theory, we calculated parameters such as node strength and efficiency to measure the importance of nodes from a local perspective and obtained the local weights of the nodes. During this period, the data was generated during the processes of matrix calculation, parameter solving and model operation. When analyzing the driving reliability of CAVs based on the fuzzy evaluation method, by determining the evaluation factor set, the evaluation set and using the improved Analytic Hierarchy Process (AHP) to determine the factor weights, we obtained the driving reliability data, and integrated it with the node importance data to obtain the node weight data of the driving loop network model of CAVs considering driving reliability.

Finally, we adopted the method of improved information entropy to evaluate the efficiency of the entire driving loop network model of CAVs. In this process, combined with the obtained node weights and driving reliability data, we calculated the information entropy of each driving loop, and then obtained the overall efficiency data of the network model.

Comments 6: The manuscript lacks a quantitative comparison with alternative modeling or evaluation methods.

Response 6: Thank you very much for pointing out the lack of quantitative comparison with alternative modeling or evaluation methods in our paper. This is indeed a current shortcoming of our research. Due to time and resource constraints at this stage, we are unable to complete the supplementation of this part in the current revision. However, we have formulated a detailed follow-up research plan and will improve this section in the future. In the upcoming research, we plan to select a variety of alternative methods widely used in the field of CAVs, such as reinforcement learning, game theory models, and deep learning-related models, etc., to conduct a comprehensive quantitative comparison with our proposed methods based on ISM and improved information entropy. We will construct multiple representative case scenarios, covering different traffic conditions, numbers of vehicles, and road conditions, etc., to ensure fair testing of various methods under the same conditions. For each case scenario, we will calculate in detail and compare the performance of different methods in key indicators such as driving loop efficiency evaluation and node weight calculation. At the same time, we will also actively refer to the latest research results in related fields and continuously optimize our comparison plan. We are well aware of the importance of improving this section for enhancing the quality of the paper and will go all out to promote the follow-up research work, hoping to provide more persuasive research results for this field in the future.

Comments 7: The number of recent references, from the last two years, is limited. Including more up-to-date references will ensure that the research is grounded in the current state of knowledge and reflects the latest developments in the field.

Response 7: Thank you for your valuable comments. We have updated the references in the paper.

Reviewer 2

Comments 1: The derivation processes of the node weight calculation (such as Formulas 7-13) and the improvement of the information entropy model (Formulas 22-26) lack detailed explanations, and the basis for the values of key parameters (such as the values of λ and *k*) is not clearly stated. It is recommended to supplement the mathematical derivations and parameter sensitivity analysis to ensure the reproducibility of the method.

Response 1: Thank you for your attention to the calculation of node weights and the improvement of the information entropy model in the paper. Regarding the derivation process of the calculation of node weights, the relevant formulas refer to multiple literatures. The literature *Identification of Key Brittleness Factors in Manufacturing Systems Based on Explanatory Structure Models and Complex Networks* [29] combines ISM with complex network theory to analyze the node weights in the network model. The method for determining the node weights of the network model proposed in this literature is highly consistent with our research. However, due to the limitation of the length of the paper, the detailed derivation process of these formulas cannot be elaborated. Nevertheless, the derivation of these formulas in the references has clear and rigorous arguments. **In section 2.2.1 of the paper**, we have cited this literature. On this basis, according to the characteristics of the driving loop network of CAVs, we have carried out targeted optimization of the modeling process.

In terms of the improvement of the information entropy method, we have drawn on the research results of the paper *System Effectiveness Evaluation Method Based on Combat Loop and Improved Information Entropy* [33], and the relevant citations have been marked **in section 2.3 of the paper**. This literature uses the improved information entropy model to evaluate the efficiency of the combat system of large surface ships. The proposed improvement ideas and methods provide key references for us to optimize the information entropy model. When constructing the model, we follow the ideas given in this literature and adjust the calculation method of information entropy to make it more suitable for the efficiency evaluation of the CAVs network.

Regarding the values of key parameters, the above two literatures, for similar research scenarios, through theoretical analysis and experimental verification, have given the reasonable value ranges and bases of the parameters. For example, the in-degree node coefficient λ = 0.6 and the correction coefficient *k* = 10.

Thank you again for your valuable comments.

Comments 2: For the key data such as the "membership degree of task requirements" in Tables 5-8, it only mentions the "fuzzy statistical method", without disclosing the sources of the specific dataset, the collection conditions, and the processing procedures.

Response 2: Thank you for your valuable comments on the data of the "membership degree of task requirements" in Table 5-8 of the paper and the relevant analysis. According to the research of *Preprint platform service performance evaluation index system construction and empirical study* [31], the fuzzy comprehensive evaluation method, with fuzzy mathematics as its theoretical foundation, realizes the transformation from qualitative evaluation to quantitative evaluation by means of the membership degree theory, and it is an extremely effective comprehensive evaluation method. We have cited this literature **in section 2.2.3 of the paper**.

When analyzing the driving reliability, we introduced the fuzzy evaluation method. As described in the paper, we have set the implementation steps of the fuzzy evaluation method in detail. In the process, according to the evaluation results of experts, we obtained the fuzzy evaluation relation matrix $R_{i}$ from the factor set $U_{i}$ to the evaluation set $P_{i}$, where $r_{ij}$ represents the membership degree of the $i-th$ influencing factor belonging to the $j-th$ evaluation, and this data is obtained through expert evaluation. Experts comprehensively evaluate each influencing factor based on their rich professional knowledge and practical experience and give the corresponding membership degree. Similarly, for the membership degree $E$ of the task requirements of the edges between nodes, we also use this method to determine it.

We hope that the above supplements and explanations can dispel your doubts about this part of the content. Thank you again for your comments.

Comments 3: The division criteria for different scenarios in Table 8 (such as "whether reliability is considered") are not clearly defined, and the descriptions of the differences in some scenarios (such as Scenario 5 and the solution in this paper) are ambiguous.

Response 3: Thank you for pointing out the problems of unclear scenario division criteria and unclear description of scenario differences in the paper. We attach great importance to this feedback and have supplemented and explained the relevant content after careful study.

**In section 3.5, Model Validation and Comparative Analysis,** we have supplemented the criteria for dividing each scenario. Scenario 1 simulates the simple driving mode of traditional vehicles, including only the basic loops of target, perception, decision, and execution. It does not consider external information interaction and focuses only on a single target, serving as a basis for comparison to highlight the unique characteristics of the CAVs network. Based on Scenario 1, in Scenario 2, the reliability of all nodes is assumed to be 1, ignoring the differences in node reliability, with the aim of exploring the influence mechanism of node reliability on efficiency evaluation. Scenario 3, on the basis of Scenario 1, introduces multiple targets, namely multiple $T1$nodes. By increasing the number of targets, it examines the changes in the efficiency of the network model when handling multiple tasks. Scenario 4 makes a more detailed division of the system in Scenario 1, that is, increasing the number of $P$ nodes, but without considering the information feedback adjustment between nodes, so as to analyze the impact of the degree of system subdivision on network efficiency. Scenario 5 further increases the number of target $T1$ nodes on the basis of Scenario 4, comprehensively studying the change rules of network efficiency under the combined effect of multiple targets and system subdivision. The scenario in this paper, on the basis of Scenario 5, fully considers information interaction, which is a key feature that differentiates CAVs from traditional vehicles. Through this setting, the network model is made to better fit the actual operation scenarios of CAVs. **In section 3.1 of the paper,** where the network model is constructed, we have added Table 2, the names of each node in the driving loop network model. By using charts, the construction of the network model is made clearer, further clarifying the considerations of each node in the scenario settings. We have also supplemented the scenario settings **in section 3.5** to make the expressions clearer.

The following is the revised content:

Scenario 1: Only the traditional driving loop network is considered, consisting of target, perception, decision, and execution cycles, without external information interaction and with only a single objective.

Scenario 2: On the basis of Scenario 1, the driving reliability is not taken into account, that is, the driving reliability equals 1.

Scenario 3: Based on Scenario 1, consider multiple objectives, that is, multiple $T$ nodes.

Scenario 4: Based on Scenario 1, the system is further subdivided, but the information feedback adjustment between nodes is not considered, that is, there are multiple $P$ nodes.

Scenario 5: Based on Scenario 4, consider adding objectives, that is, multiple $T$ and $P$ nodes.

This paper scenario: Based on Situation 5, consider the information interaction, that is, add the information set $I$ module.

The following is the added Table 2:

**Table 2. Symbols of each node in the driving loop network model of CAVs and the modules they represent.**

| Modules | Node symbols | Node names |
| --- | --- | --- |
| $T$(Target) | $T1$ | Safe collision avoidance |
|  | $T2$ | Optimal path |
| $P$(Perception) | $P1$ | Perception system |
|  | $P2$ | Communication collaboration system |
| $I$(Information) | $I1$ | Personal information |
|  | $I2$ | Surrounding environment information |
| $D$(Decision) | $D1$ | Global decision planning |
|  | $D2$ | Local decision planning |
| $E$(Execution) | $E$ | Execution control |

Comments 4: The study only verifies the method based on a single-objective-driven model (Figures 5-7), and does not involve the robustness tests under complex road conditions (such as sudden obstacles and multi-vehicle coordination).

Response 4: Thank you very much for pointing out the limitations in the verification of the research methods in our paper, namely, the verification is only based on a single - target - driven model, and there is a lack of robustness tests under complex road conditions, such as sudden obstacle and multi - vehicle coordination scenarios. We are deeply aware that this issue is crucial to the integrity and reliability of our research.

Currently, due to time and resource constraints, we are unable to supplement this part of the content in this submission. However, we have already formulated a detailed future research plan to improve this section. In subsequent research, we will focus on the robustness testing of the research methods proposed in this paper under complex road scenarios.

For the sudden obstacle scenario, we plan to use a high - precision vehicle dynamics simulation platform combined with actual road test data to simulate various situations of obstacle appearance, including different obstacle shapes, sizes, positions, and speeds. Through these simulations, we will apply the proposed method to evaluate the efficiency of the driving loop network, observe the response speed and accuracy of the vehicle decision - making system, as well as the stability of the entire network model when dealing with sudden situations.

In terms of the multi - vehicle coordination scenario, we will construct a complex traffic model of multi - vehicle interaction, covering different traffic flows, road layouts, and vehicle driving rules. Using this model, we will test whether our method can accurately evaluate the efficiency of the driving loop during multi - vehicle coordinated driving, and whether it can effectively coordinate the actions of vehicles to avoid collisions and traffic congestion.

To ensure the reliability and scientific nature of the test results, we will also establish a strict evaluation index system, including multiple aspects such as vehicle driving safety, efficiency, and energy consumption. Through these comprehensive tests and analyses, we hope to gain an in - depth understanding of the performance of the research methods proposed in this paper under complex road conditions, and further improve the research on the construction of CAVs network models and the evaluation of driving loop efficiency. Thank you again for your valuable comments. We look forward to contributing more comprehensive and reliable research results to this field in the future.

Comments 5: The analysis of the current situation of the research on the CAVs network model in the introduction section is rather general, and it does not deeply compare the advantages and disadvantages between the existing methods (such as reinforcement learning and game theory models) and the ISM method in this paper. It is necessary to expand the literature review and highlight the irreplaceability of the innovation points of this paper.

Response 5: Thank you for your valuable comments on the literature review in the introduction section of the paper. In response to your suggestions, we have reorganized the literature review section, starting from the network modeling of vehicles, then moving on to the efficiency evaluation of vehicles, and finally the research on node importance. Through the reorganization of the literature review, we have identified the current research deficiencies regarding CAVs: Most previous studies have focused on network modeling of traditional vehicles or electric vehicles. In terms of efficiency evaluation, the assessments mainly focus on some aspects of vehicle performance during driving, such as stability performance and dynamic performance, from a microscopic perspective. Few scholars have comprehensively considered the node importance from both the global and local aspects of the driving network for CAVs, nor have they taken into account the reliability of the driving system itself, resulting in incomplete evaluation results. Therefore, we have clarified the purpose of this study and pointed out the innovations of this paper.

**References**

1. Yang L, Li H. Vehicle‐to‐vehicle communication based on a peer‐to‐peer network with graph theory and consensus algorithm[J]. IET Intelligent Transport Systems, 2019, 13(2): 280-285.
2. Ma J, Cheng L, Li D, et al. Stochastic electric vehicle network considering environmental costs[J]. Sustainability, 2018, 10(8): 2888.
3. Tu Q, Li M, Wu Y. A Reliability-Based Network Equilibrium Model with Electric Vehicles and Gasoline Vehicles[J]. Promet-Traffic&Transportation, 2024, 36(1): 83-99.
4. Xu Q, Chen C, Chang X, et al. Modeling and analysis of mixed traffic networks with human-driven and autonomous vehicles[J]. Chinese Journal of Mechanical Engineering, 2024, 37(1): 134.
5. Zhao J, Liu J, Yang L, et al. Exploring the relationships between subjective evaluations and objective metrics of vehicle dynamic performance[J]. Journal of Advanced Transportation, 2018, 2018(1): 2638474.
6. Wang B, Huang W, Lu B, et al. A novel prediction and evaluation method for vehicle stability[J]. International Journal of Vehicle Design, 2024, 95(3-4): 205-221.
7. Huang W L, Wen D, Geng J, et al. Task-specific performance evaluation of UGVs: Case studies at the IVFC[J]. IEEE transactions on intelligent transportation systems, 2014, 15(5): 1969-1979.
8. Wang W, Li W B, Qu F F, et al. An improved grey relational theory evaluation method: considering the comprehensive performance of autonomous vehicles in virtual test[J]. International Journal of Automotive Technology, 2024, 25(5): 1235-1249.
9. Park J Y, Heo S J, Kang D O. Development of torque vectoring control algorithm for front wheel driven dual motor system and evaluation of vehicle dynamics performance[J]. International Journal of Automotive Technology, 2020, 21: 1283-1291.
10. Huang X, Chen J, Cai M, et al. Traffic node importance evaluation based on clustering in represented transportation networks[J]. IEEE Transactions on Intelligent Transportation Systems, 2022, 23(9): 16622-16631.
11. Zhang Chen, Liang Yichen, Peng Peng, et al. Xi 'an subway network node importance evaluation [J/OL]. Railway standard design, 1-9 [2025-04-09]. https://doi.org/10.13238/j.issn.1004-2954.202404010013. (In Chinese)
12. Feng Juntai, Qian Yongsheng, Zeng Junwei, et al. Layout method of trunk highway network based on terrain factors [J]. Highways, 2019,70(03):223-229. (In Chinese)
13. Mi X, Shao C, Dong C, et al. A framework for intersection traffic safety screening with the implementation of complex network theory[J]. Journal of advanced transportation, 2020, 2020(1): 8824447.
14. Xu M, Wu J, Liu M, et al. Discovery of critical nodes in road networks through mining from vehicle trajectories[J]. IEEE Transactions on Intelligent Transportation Systems, 2018, 20(2): 583-593.
15. Zhang Y, Zheng S, Chen Y. Identification of key nodes in comprehensive transportation network: A case study in Beijing-Tianjin-Hebei urban agglomeration, China[J]. Transportation Research Record, 2024, 2678(5): 827-840.
16. Zheng Wenping, Wu Zhikang, Yang GUI. A key node identification Algorithm based on local centrality [J]. Journal of Computer Research and Development,2019,56(09):1872-1880. (In Chinese)
17. Wang Jianwei, Rong Lili, Guo Tianzhu. A method for measuring network node importance based on local features [J]. Journal of Dalian University of Technology,2010,50(05):822-826. (In Chinese)
18. Hu Gang and Wang Qin. Importance identification of complex network nodes based on cross-layer information entropy [J]. Journal of Xihua University (Natural Science), 2019,44(02):70-78. (In Chinese)
19. Zhang X, Zhang W, Lee P T W. Importance rankings of nodes in the China Railway Express network under the Belt and Road Initiative[J]. Transportation Research Part A: Policy and Practice, 2020, 139: 134-147.
20. Xu Huanjun, Yu Jing, Yuan Shuai, et al. Research on Taxi stop location selection algorithm based on POI [J]. High-tech Communication,2021,31(11):1154-1163. (In Chinese)
21. You Qianjing, Zheng Wei, Liu Fangli. Node importance evaluation based on overlapping box cover algorithm [J]. Computer application research, 2021, 38 (11): 3354-3358. The DOI: 10.19734 / j.i SSN. 1001-3695.2021.04.0102. (In Chinese)
22. Qureshi, K.N., Din, S., Jeon, G., Piccialli, F., 2021. Internet of Vehicles: Key Technologies, Network Model, Solutions and Challenges With Future Aspects. IEEE Transactions on Intelligent Transportation Systems 22 3, 1777–1786. doi:https://doi.org/10.1109/tits.2020.2994972
23. Jiang, D., Huo, L., Zhang, P., Lv, Z., 2021. Energy-Efficient Heterogeneous Networking for Electric Vehicles Networks in Smart Future Cities. IEEE Transactions on Intelligent Transportation Systems 22 3, 1868–1880. doi:https://doi.org/10.1109/tits.2020.3029015
24. Stepanyants, V.G., Romanov, A.Y., 2023. A Survey of Integrated Simulation Environments for Connected Automated Vehicles: Requirements, Tools, and Architecture. IEEE Intelligent Transportation Systems Magazine 16 2, 6–22. doi:https://doi.org/10.1109/mits.2023.3335126
25. Wang, L., Zhang, X., 2023. Anomaly Detection for Automated Vehicles Integrating Continuous Wavelet Transform and Convolutional Neural Network. Applied Sciences 13 9, 5525. doi:https://doi.org/10.3390/app13095525
26. Pal, P., Gopal, P.R.C., Ramkumar, M., 2023. Impact of transportation on climate change: An ecological modernization theoretical perspective. Transport Policy 130, 167–183. doi:https://doi.org/10.1016/j.tranpol.2022.11.008
27. Shi, Q., Yu, T., Zuo, J., Lai, X., 2016. Challenges of developing sustainable neighborhoods in China. Journal of Cleaner Production 135, 972–983. doi:https://doi.org/10.1016/j.jclepro.2016.07.016
28. Cheng Mengjie, Yuan Lingyun Construction of Chinese Bridge Knowledge System Based on ISM and Node Degree [J]. Journal of Yunnan Normal University (Natural Science Edition),2022,42(03):75-78.
29. Liu Weiqiang, Xu Liyun. Identification of Key Brittleness Factors in Manufacturing Systems Based on Explanatory Structure Models and Complex Networks [J] Computer integrated manufacturing system, 2021, 27 (11) : 3076-3092. The DOI: 10.13196 / j.carol carroll ims 2021.11.003.
30. Wu Leilei. Several kinds of common features of traffic network differentiation power law research [D]. Beijing jiaotong university, 2021. The DOI: 10.26944 /, dc nki. Gbfju. 2021.002794.
31. Zhou Yifu, Tan Chunhui, Zhou Yang, et al. Preprint platform service performance evaluation index system construction and empirical study [J]. Journal of library intelligence, 2025,69 (02): 56-66. The DOI: 10.13266 / j.i SSN. 0252-3116.2025.02.006.
32. Li, F., Phoon, K.K., Du, X., Zhang, M., 2013. Improved AHP Method and Its Application in Risk Identification. Journal of Construction Engineering and Management 139 3, 312–320. doi:https://doi.org/10.1061/(asce)co.1943-7862.0000605
33. Luo Chengkun, Chen Yunxiang, Wang Lili, et al. System Effectiveness Evaluation Method Based on Combat Loop and Improved Information Entropy [J]. Systems Engineering and Electronic Technology,2019,41(01):73-80.

We tried our best to improve the manuscript and made some changes in the manuscript. These changes will not influence the content and framework of the paper. We appreciate for Editor/Reviewers’ warm work, and hope the correction will meet with approval. Once again, thank you very much for your comments and suggestions.

Sincerely,

Daowen Zhang.
